# High-Intensity Resistance Training Enhances Strength, But Not Muscle Mass and Physical Functioning Gains during Total Knee Arthroplasty Rehabilitation

**DOI:** 10.1249/MSS.0000000000004003

**Published:** 2026-04-07

**Authors:** ALEJANDRA P. MONSEGUE, ESTHER J. C. WILMS, CHARLOTTE S. KRAMER, JORDI MORWANI-MAGNANI, MYRTHE VAN DER HAIJDEN, CECILE M. SINGH-POVEL, POL GROOTSWAGERS, LISETTE C. P. G. M. DE GROOT, P. ELINE SLAGBOOM, LUC J. C. VAN LOON, LEX B. VERDIJK

**Affiliations:** 1 Department of Human Biology, NUTRIM, Maastricht University Medical Centre+, Maastricht, the Netherlands; 2 Division of Human Nutrition and Health, Wageningen University & Research, Wageningen, the Netherlands; 3 Leiden University Medical Centre, Leiden, the Netherlands; 4 FrieslandCampina, Amersfoort, the Netherlands

**Keywords:** KNEE REPLACEMENT, PHYSIOTHERAPY, STRENGTH TRAINING, SURGERY

## Abstract

**Purpose:**

To determine whether high-intensity resistance exercise training with nutritional support (RET) during recovery from total knee arthroplasty induces greater improvements in muscle-related outcomes compared with standard rehabilitation (SR) alone.

**Methods:**

Thirty-three patients (70 ± 6 yr; 28.7 ± 3.1 kg⋅m^−2^) were randomized to receive RET (intervention + regular rehabilitation, *n* = 18) or SR (regular rehabilitation only, *n* = 15) for 12 wk, starting 8 wk post-TKA. RET involved supervised, bilateral, high-intensity resistance exercise, 3×/wk, daily supplementation (45 g protein, 5.5 g Vivinal GOS, 800 IU vitamin D, 366 mg calcium), and dietary counseling. Outcomes included bilateral and unilateral leg press one-repetition maximum, DXA-derived appendicular lean mass, computed tomography-derived quadriceps cross-sectional area, 6-min walking test, and 5-times chair-stand test (5CST). Data are mean ± SD, analyzed with two-way repeated-measures ANOVAs, or median [IQR], analyzed with Wilcoxon Signed-Rank test.

**Results:**

Bilateral leg press one-repetition maximum improved to a greater extent following RET (131 ± 38 to 174 ± 56 kg; *P* < 0.001) versus SR (124 ± 36 to 143 ± 49 kg, *P* = 0.018, *P* value of interaction [*P*_int_] = 0.026). Strength in the nonoperated leg increased in the RET group only (RET: 22% ± 17%, *P* < 0.001, SR: 6% ± 11%, *P* = 0.175, *P*_int_ = 0.002). Operated leg strength increased similarly between groups (RET: 51% ± 33%, SR: 40% ± 30%, *P* value of main time effect [*P*_time_] < 0.001, *P*_int_ = 0.338), as did appendicular lean mass (RET: 0.5 ± 0.8 kg, SR: 0.3 ± 0.8 kg, *P*_time_ = 0.009, *P*_int_ = 0.390), and quadriceps cross-sectional area (operated: RET: 7.8% ± 7.5%, SR: 9.2% ± 5.9%, nonoperated: RET: 4.8% ± 4.5%, SR: 3.8% ± 3.7%, *P*_time_ < 0.001, *P*_int_ ≥ 0.557). Six-minute walking test improved more in the SR (428 ± 94 to 513 ± 75 m, *P* < 0.001) versus RET group (417 ± 69 to 460 ± 72 m, *P* = 0.002, *P*_int_ = 0.034). 5CST only improved significantly following RET (RET: 15.3 [4.2] to 13.2 [3.9] s, *P* = 0.039, SR: 14.4 [4.0] to 14.4 [5.1] s, *P* = 0.064).

**Conclusions:**

Compared with standard total knee arthroplasty rehabilitation, high-intensity resistance training with nutritional support induces greater gains in bilateral strength but not muscle mass or physical functioning.

The age-related loss of muscle mass and strength is associated with a broad range of negative health consequences, including musculoskeletal, immuno-metabolic, and cognitive impairments (1). Affecting over 20% of adults over the age of 40, knee osteoarthritis is a degenerative disease that exacerbates muscle loss as patients become physically compromised by pain and impaired joint mobility (2). Despite total knee arthroplasty (TKA) being the gold standard treatment for end-stage knee osteoarthritis, TKA patients remain with deficits in physical functioning, muscle mass, and muscle strength compared with healthy counterparts, even up to years after successful surgery (3–5). Therefore, addressing these muscle deficits in TKA patients is of critical importance.

Physical rehabilitation is crucial for patient recovery following TKA. Although the methods and equipment employed by physical therapists during TKA rehabilitation vary widely, it is common practice to primarily work toward recovering joint mobility and physical functioning in daily life. Improving muscle strength is generally only a secondary goal, and increasing muscle mass is rarely a direct focus. Consequently, muscle-strengthening exercises performed as part of standard rehabilitation (SR) practices tend to be conducted at relatively low intensities, e.g. using bodyweight exercises with little to no external loads (6).

In contrast to low-intensity exercise, high-intensity, progressive resistance exercise training leads to greater increases in muscle strength (7), and is even effective in increasing physical functioning in compromised populations (8,9). It has therefore been suggested that high-intensity resistance exercise training be incorporated into physical rehabilitation regimes following TKA (6). However, the available literature investigating the effects of high-intensity resistance exercise-based TKA rehabilitation compared with standard TKA rehabilitation is limited. Moreover, the studies performed so far vary widely in their applied methodology regarding e.g. training intensities, frequency, volume, equipment, and so on (10). Considering the importance of these basic training principles in determining training response, it is unsurprising that the results of these studies are also highly heterogeneous, with some studies showing greater improvements in muscle mass, strength, and functional outcomes following high-intensity resistance exercise-based rehabilitation compared with SR (11–17), while others show no differences (18–21).

Upon critical evaluation of the existing literature, we have recently argued that resistance exercise-based rehabilitation is likely superior to standard care for TKA patients when exercises are performed at a high intensity (70%–80% of the one-repetition maximum), with sufficient volume (3–4 sets per exercise, preferably 3 times/wk), for at least 8 wk (10). In addition to optimizing the physical rehabilitation protocol, ensuring adequate nutrition is essential for supporting muscle adaptations and promoting optimal recovery from exercise training (22–24). This specifically includes sufficient intake of protein and key micronutrients that have been implicated in muscle health, such as vitamin D and calcium (25–28). Therefore, in the present study, we assessed the effects of 12 wk of high-intensity resistance exercise training with nutritional support compared with only SR on muscle-related rehabilitation outcomes in older adults recovering from TKA. We hypothesized that an optimized, combined exercise and nutritional intervention would be superior to SR alone in improving muscle mass (primary outcome) as well as muscle strength and physical functioning.

## METHODS

### Study Design

**FIGURE 1:**
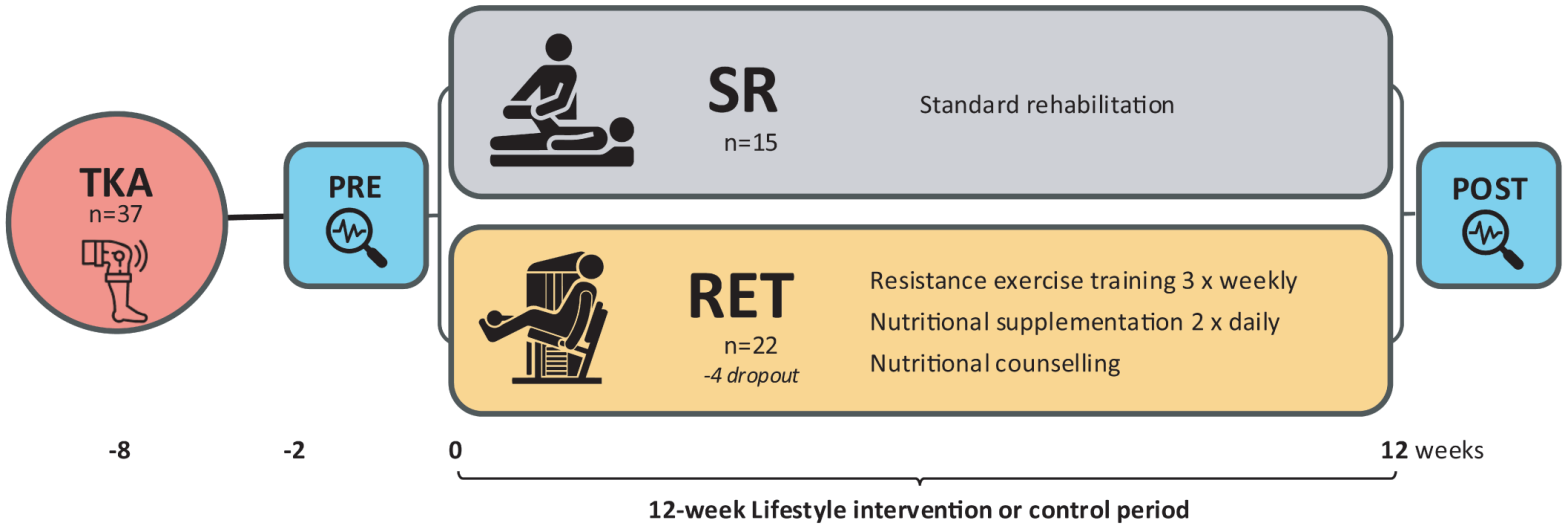
Study timeline. The 12-wk lifestyle intervention or control period began 7–9 wk following surgery. Outcome measures were assessed before (pre) and after (post) this period. Post-testing of the questionnaires, habitual dietary intake and physical activity were assessed in the final week of the intervention or control period.

This trial is part of a larger study investigating the effects of a lifestyle intervention (high-intensity resistance exercise training supported by nutritional supplementation and dietary counseling) on health outcomes in different groups of health-compromised older adults (25). The present study involves the knee replacement cohort from this larger multi-center trial (25), which was approved by the Medical Research Ethics Committee Leiden Den Haag Delft, the Netherlands (P21.049), and complied with the guidelines of the World Medical Association Declaration of Helsinki. Patients who were planned to undergo or had recently undergone TKA received written and verbal information about the study, including all procedures and potential risks. Interested patients gave their informed consent and were screened on eligibility criteria. The screening session was performed around 5–6 wk post-TKA owing to feasibility reasons, aligning with when most patients were allowed to walk without crutches and drive independently. Once eligibility was granted by the study’s medical doctor, included patients were randomly allocated into a lifestyle intervention group or a control group. All participants were encouraged to continue their rehabilitation as directed by their own treating physician/physiotherapist throughout the entire study duration. Baseline assessments were performed between 6 and 8 wk post-TKA, after which the 12-wk intervention/control period began. During this period, patients in the intervention group started with a lifestyle intervention consisting of progressive, high-intensity resistance exercise training with nutritional support (RET group), while the control group continued to only receive their SR group. Outcomes were assessed again within 1 wk following the final exercise session. Figure 1 displays the study timeline.

### Participants

**FIGURE 2:**
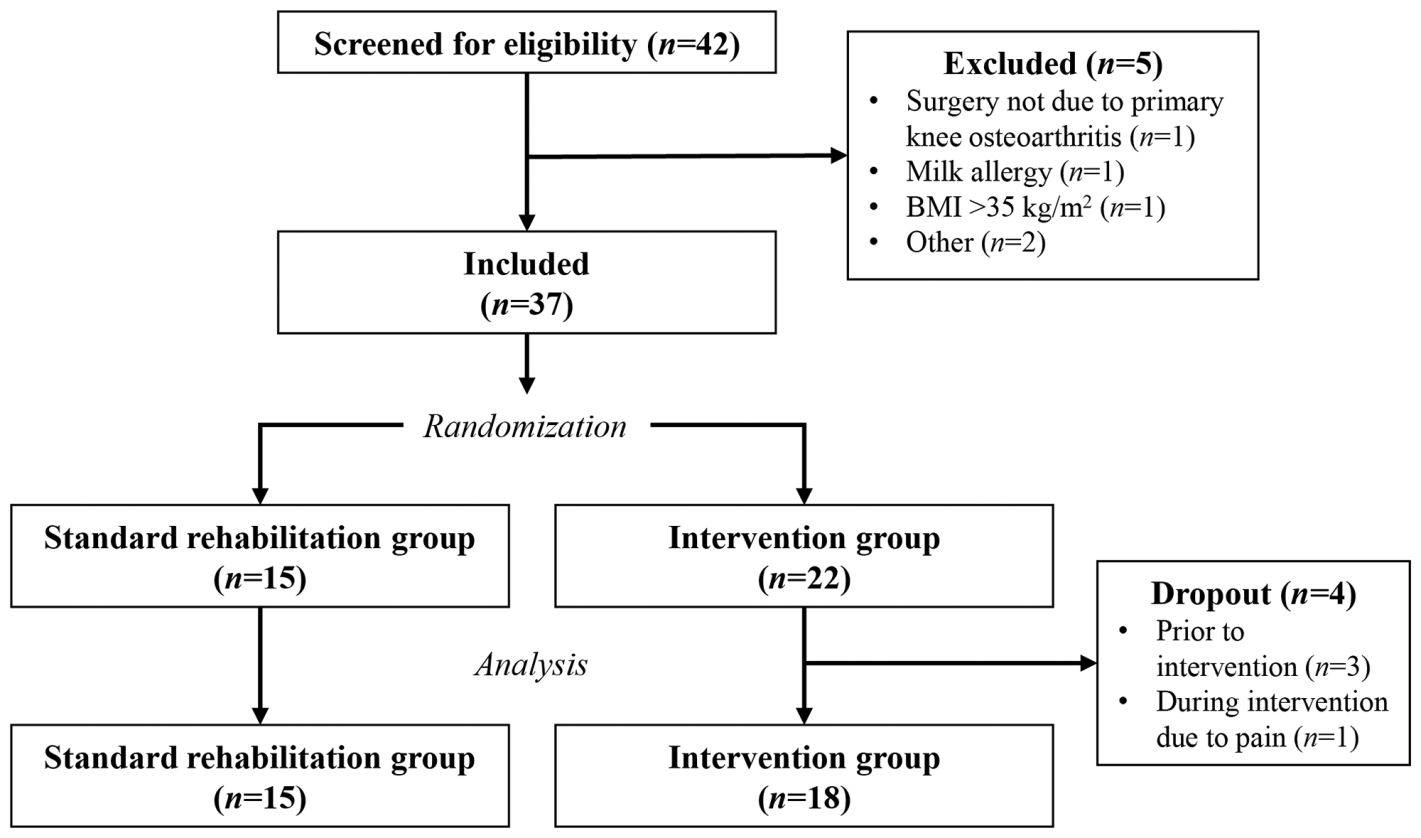
Flow diagram of participant inclusion, group allocation, and analysis.

Thirty-seven patients aged ≥60 years with a body mass index between 18.5 and 35 kg⋅m^−2^ who underwent a primary TKA owing to knee osteoarthritis participated in the present study. A flow diagram of participant allocation is presented in Figure 2. Four patients in the RET group withdrew participation, three of which did so before starting the intervention period and one participant dropped out during the intervention owing to persistent pain in the knee. Participant characteristics are displayed in Table 1. During the screening visit, participants were assessed for eligibility. Participants were excluded if they had any known medical condition that could influence participant safety and study results, including contraindication for high protein intake or dietary restrictions on milk/prebiotics/vitamin D/calcium (e.g., an estimated glomerular filtration rate <30 mL⋅min^−1^⋅1.73 m^−2^ or severe gastrointestinal conditions/procedures), and contraindications for high-intensity exercise (e.g., severe or uncontrolled cardiorespiratory conditions, such as a resting blood pressure > 160/100 mmHg, COPD > stage I, uncontrolled dysrhythmias, severe stenotic or regurgitant valvular disease, hypertrophic cardiomyopathy, or peripheral artery disease > Fontaine II). Participants were also excluded if they had active cancer or collagen disorders. Additionally, participants needed to be community dwelling and mentally competent (i.e., a Mini-Mental State Examination score of ≥24) and should not have been following a structured high-intensity exercise program in the year before study participation.

**TABLE 1.** Participant baseline characteristics.

	RET	SR
*n* (F)	18 (7)	15 (7)
Age, yr	72 ± 5	68 ± 6
Height, m	172 ± 10	171 ± 7
Weight, kg	83 ± 10	85 ± 12
BMI, kg·m^−2^	28 ± 3.2	29 ± 3.0
Waist circumference, cm	99 ± 5.8	101 ± 7.9
BP systolic, mmHg	133 ± 14	138 ± 15
BP diastolic, mmHg	75 ± 10	78 ± 9
HR rest, bpm	76 ± 10	71 ± 8

Data are mean ± SD.

BMI, body mass index; BP, blood pressure; HR, heart rate.

### Standard Rehabilitation

All study participants were encouraged to continue following the rehabilitation practices provided/recommended by their treating physician and physiotherapist. As TKA rehabilitation is highly variable across physiotherapy practices and per individual patient, differing in exercises, load intensities, and frequency of supervised sessions, a short questionnaire was administered at the end of the study in order to quantify the frequency and describe the nature of the supervised physiotherapy that each patient received during the study period. While patients in the SR group only received their SR, patients in the RET group additionally took part in the lifestyle intervention involving high-intensity resistance exercise training with nutritional support.

### Lifestyle Intervention

The RET group underwent 12 wk of high-intensity full-body resistance exercise training, which was supported by nutritional supplementation and dietary advice. The nutritional supplements were consumed every morning (25 g whey protein [Nutri Whey Isolate], 5.5 g Vivinal GOS, 800 IU vitamin D, 250 mg calcium, 153 kcal) and evening (20 g whey protein, 116 mg calcium, 85 kcal), in the form of powders that were dissolved in ~100 mL water. Apart from protein, supplement composition was based on the proposed benefits of vitamin D, calcium, and fiber for (muscle) health (25–27). We specifically chose to combine nutritional support with the high-intensity resistance training program, to create an optimized intervention for inducing muscular adaptations in a compromised patient population. In line with recent evidence, we therefore selected a 12-wk intervention period, with daily provision of 40–50 g additional protein, calcium, and 800 IU vitamin D (28). Consumption of the supplements was logged in a diary that was returned to the researchers every 2 wk. At baseline and half-way through the intervention period, dietary advice was provided based on the results of the Eetscore, a questionnaire evaluating the extent to which one eats in accordance with the Dutch Dietary Guidelines 2015 (29).

For the exercise training component, training sessions were performed bilaterally on standard weight-lifting machines under supervision of a trained researcher. Exercise sessions (45–60 min in duration) were performed 3 times weekly, with at least 1 d rest in between sessions, and included a 5-min warm-up on a cycle ergometer at a resistance of ~50 W, followed by 7 exercises (leg press, chest press, horizontal row, calf raises, shoulder press, vertical pull, and leg extension). Over the first 3 wk, exercise intensity progressed from ~50% to ~70% of the one-repetition maximum (1RM), with each exercise being performed for 2 (upper-body) or 3 (lower-body) sets of 10–15 repetitions, with at least 1.5-min rest between all sets and exercises. From week 4, all exercises were performed at a goal intensity of 80% 1RM (adjusted based on individual patient progression) for 8 repetitions per set, and the number of sets for the leg press and leg extension were increased to 4 per exercise. The final set of each exercise was taken to voluntary failure from week 2 onwards. In order to ensure progressive overload, exercise load was increased by 2.5–5 kg in the subsequent session if the participant achieved >10 repetitions in the final set of an exercise. Additionally, the 1RM on each exercise was reassessed after 4 and 8 wk of training so that the goal intensity of 80% 1RM could be ensured. Calf raises were performed for 2 sets of 15–20 repetitions throughout the entire study duration. Furthermore, the calf raise exercise was not taken to voluntary failure nor assessed for 1RM. However, progressive overload was guaranteed by incrementally increasing the load at each training at the discretion of the trainer.

### Anthropometrics

Body height and mass, and waist circumference were assessed in the morning in a fasted state with the participant wearing only light clothing and no shoes. Height and body mass were assessed on a stadiometer to the nearest 0.5 cm and on a digital weighing scale to the nearest 0.1 kg, respectively. Using a measuring tape, waist circumference was measured twice in succession on bare skin at the midpoint between the lowest rib and the iliac crest, to the nearest 0.5 cm. The waist circumference was determined as the average of the two measurements (with a difference no larger than 1 cm).

### Body Composition and Quadriceps Cross-sectional Area

In a fasted state, whole body and regional lean and fat mass were assessed using dual energy X-ray absorptiometry (DXA; Discovery A; Hologic). As a proxy for muscle mass, the cross-sectional area (CSA) of the quadriceps muscles was determined using single-slice computed tomography (CT; Somatom Definition Flash; Siemens Healthineers, Forchheim, Germany) as previously described (30). Briefly, while lying in a supine position, a 2-mm thick axial image was taken of the upper thighs at a distance of 15 cm proximal to the top of the patella of the nonoperated knee. CT images were analyzed in a blinded fashion through tracing of the quadriceps and whole-thigh muscles on ImageJ software.

### Muscle Strength

Muscle strength was assessed by 1RM testing on all exercise machines; only the leg press and leg extension 1RMs are presented as outcomes in the current investigation. Patients first participated in a familiarization session during which they were instructed on proper technique for each exercise, and an estimation of their 1RM was determined using the multiple repetitions testing procedure (31). After a minimum of 2 d following the familiarization session (baseline testing) or last training session (post-testing), the bilateral 1RM was determined for each exercise, as described previously (32). Briefly, after a general warm-up of 5 min on a cycle ergometer, each exercise began with a warm-up set of 10 repetitions at a load equivalent to 50% of the estimated 1RM, followed by a second warm-up set of five repetitions at 75% of the estimated 1RM. Thereafter, the load was increased until the maximal possible weight was achieved to the nearest 2.5 kg, with 2 min rest in between attempts. Participants were verbally encouraged throughout the 1RM tests to ensure maximal effort. Following the bilateral leg press and leg extension 1RMs, unilateral 1RMs were also tested on those machines in order to determine muscle strength in the operated and nonoperated legs separately. After 5-min rest, the nonoperated leg was tested first, followed by the operated leg. Procedures were similar to the bilateral 1RMs. The order of 1RM assessments was repeated at the post measurements.

### Physical Functioning

Just before the 1RM assessments, physical functioning was measured through a variety of tests, including the Short Physical Performance Battery (SPPB) (33), timed up-and-go (TUG) test (34), and 6-min walk test (6MWT) (35). The SPPB consists of three components (balance, gait speed, and chair-stand test), for which a maximum of four points per component can be awarded based on established cutoff values (33). The balance component involves standing in three progressively difficult stances (feet together, semitandem, and full tandem) held for up to 10 s, with time measured to the nearest 0.01 s. Gait speed was evaluated as the time required to walk 4 m at a usual, self-selected walking pace. The chair-stand test assesses the time required to rise from a chair (seat height: 45 cm) 5 times as quickly as possible without arm assistance. The total SPPB score, as well as the time taken to complete the chair-stand test (5CST) were included in the analyses. If a participant could not perform the 5CST, a value of 60 s was used in the analyses. The TUG assesses the time needed for a participant to rise from a chair, walk 3 m, turn around, and return to sit on the starting chair. For the 6MWT, participants walked back and forth around two cones separated by a distance of 30 m as quickly as possible without use of a walking aid. The distance covered after exactly 6 min was recorded to the nearest 1 m.

### Questionnaires

Qualitative health outcomes were assessed via questionnaires at baseline and during the last week of the 12-wk intervention/control period. These included the Western Ontario and McMaster Universities Arthritis Index (WOMAC) (36), the 36-item short-form health survey (SF-36) (37), and the short-form Sarcopenia Quality of Life (SF-SarQoL) (38) questionnaires. The WOMAC assesses TKA-specific functional outcomes and is composed of three domains (pain, stiffness, and physical functioning). The SF-36 contains questions that cover eight different items (physical functioning, role limitations owing to physical health, role limitations owing to emotional problems, vitality, mental health, social functioning, pain, and general health). The SF-SarQoL is a measure of sarcopenia-related quality of life. The WOMAC total score and all subscores, the SF-36 physical functioning, role limitations owing to physical health, and pain scales, and the total score for the SF-SarQoL were analyzed for the current investigation.

### Dietary Intake and Physical Activity

Habitual energy and macronutrient intake were assessed via 3-d food diaries, which were recorded at baseline and during the last week of the 12-wk intervention/control period. The 3 d on which dietary intake was recorded were randomized over the entire cohort to get a balanced distribution of recorded days. Dietary data were processed in Compleat (Human Nutrition WUR, Wageningen, NL), and habitual energy and macronutrient intake were determined as the averages over the three recorded days. During those same weeks, physical activity was recorded by means of a three-axial accelerometer (Actigraph GT3x-BT) with a sampling rate of 30 Hz, which was worn at the hip for seven consecutive days during waking hours. The total metabolic equivalent of task (MET) and average daily activity-related energy expenditure, steps, and hours worn were extracted. Days on which the accelerometer was worn for at least 90% of awake time were deemed suitable for analyses. Additionally, a physical activity diary was kept over the 7 d as a means of accounting for any extreme values in the accelerometer output.

### Statistical Analyses

Sample size calculations were performed for the primary outcome (quadriceps muscle CSA) as well as the main secondary outcomes (1RM leg press, 5CST). Based on previous data (12,13,39,40), we expected improvements to be twice as large in the RET versus SR group, resulting in an estimated effect size of 1.05 (1RM, 5CST) up to 1.2 (quadriceps CSA). Taking the lower boundary, a total sample size of *n* = 32 was calculated.

Data are expressed as mean ± SD, unless otherwise specified, and were analyzed using statistical software SPSS (version 24.0; IBM Corporation, Armonk, NY), with significance set at *P* < 0.05. Changes over time between groups were analyzed with two-factor repeated-measures ANOVA tests, with time (baseline vs post-testing) as the within-subjects factor and group (RET vs SR) as the between-subjects factor. In the case of a significant interaction effect, simple effects of time (within each group) and group (at each time point) are reported. For the 5CST, data are presented as median [IQR], and nonparametric tests were applied; within-group changes over time were assessed separately for each group using Wilcoxon Signed-Rank tests, and between-group differences were assessed by performing a Mann–Whitney *U* test on the change scores (post minus pre). Effect sizes are reported as partial eta squared (η^2^) where appropriate.

## RESULTS

### Participant Characteristics and Intervention Compliance

Table 1 shows participant characteristics at baseline. The RET group (*n* = 18) consisted of 61% males, whereas the SR group (*n* = 15) had 53% males. Participants were overweight, with an average body mass index of 28.7 ± 3.1 kg⋅m^−2^. Participants in the RET group completed 97% ± 6% of the training sessions (range: 81%–100%) and consumed 94% ± 4% of the supplements (range: 90%–100%). In addition to the training sessions, participants in the RET group attended a median of two physiotherapy sessions (interquartile range: 0–8 sessions) during the 12-wk intervention period, whereas the SR group attended a median of 12 physiotherapy sessions (interquartile range: 8–20 sessions). Apart from bodyweight-based exercises (both supervised and unsupervised), 0 RET and 4 SR participants reported performing resistance exercises using additional weights with their physiotherapist, all reporting low exercise loads.

### Body Composition and Quadriceps Cross-sectional Area

One participant in the RET group had a pacemaker that was contraindicated for the DXA scan; body composition data are available for the remaining participants (SR: *n* = 15, RET: *n* = 17) and are presented in Table 2. Although total body mass did not change significantly over time for either group (*P*_int_ = 0.145, *P*_time_ = 0.162), whole body lean mass increased significantly over time (SR: +0.3 ± 1.5 kg, RET: +1.2 ± 1.5 kg; *P* = 0.008, η^2^ = 0.21), with no significant differences between the groups (*P*_int_ = 0.085, η^2^ = 0.10). Similarly, appendicular lean mass increased by 0.3 ± 0.8 kg and 0.5 ± 0.8 kg (*P*_time_ = 0.009, η^2^ = 0.21, *P*_int_ = 0.390, η^2^ = 0.03) in the SR and RET groups, respectively. While lean mass in the nonoperated leg increased over time (*P*_time_ = 0.024, *P*_int_ = 0.611), no significant changes were observed in the operated leg (*P*_time_ = 0.937, *P*_int_ = 0.449). Total body fat percentage did not significantly change over time in either group (*P*_time_ = 0.080, *P*_int_ = 0.983).

**TABLE 2.** Body composition assessed by dual energy X-ray absorptiometry before (pre) and following (post) 12 wk of SR or a RET, which began 8 wk following TKA.

		RET (*n* = 17)	SR (*n* = 15)		*P*	
				T*G	T	G
Whole body lean mass, kg	Pre	55.0 ± 10.4	55.8 ± 11.2	0.085	**0.008**	0.940
	Post	56.3 ± 10.0	56.1 ± 11.4			
Appendicular lean mass, kg	Pre	23.7 ± 5.0	23.4 ± 5.1	0.390	**0.009**	0.804
	Post	24.2 ± 5.1	23.6 ± 5.4			
Nonoperated leg lean mass, kg	Pre	8.6 ± 1.6	8.6 ± 1.7	0.611	**0.024**	0.962
	Post	8.8 ± 1.6	8.7 ± 1.9			
Operated leg lean mass, kg	Pre	9.1 ± 1.8	8.8 ± 1.7	0.449	0.937	0.700
	Post	9.0 ± 1.8	8.8 ± 1.7			
Total body fat, %	Pre	31.7 ± 9.7	32.9 ± 7.7	0.983	0.080	0.717
	Post	31.3 ± 9.5	32.4 ± 8.1			

Data were analyzed with two-way repeated-measures ANOVA and are presented as mean ± SD. *P* values for the time*group interaction effect (T*G) and main effects of time (T) and group (G) are shown.

Significant *P* values are bolded.

**FIGURE 3:**
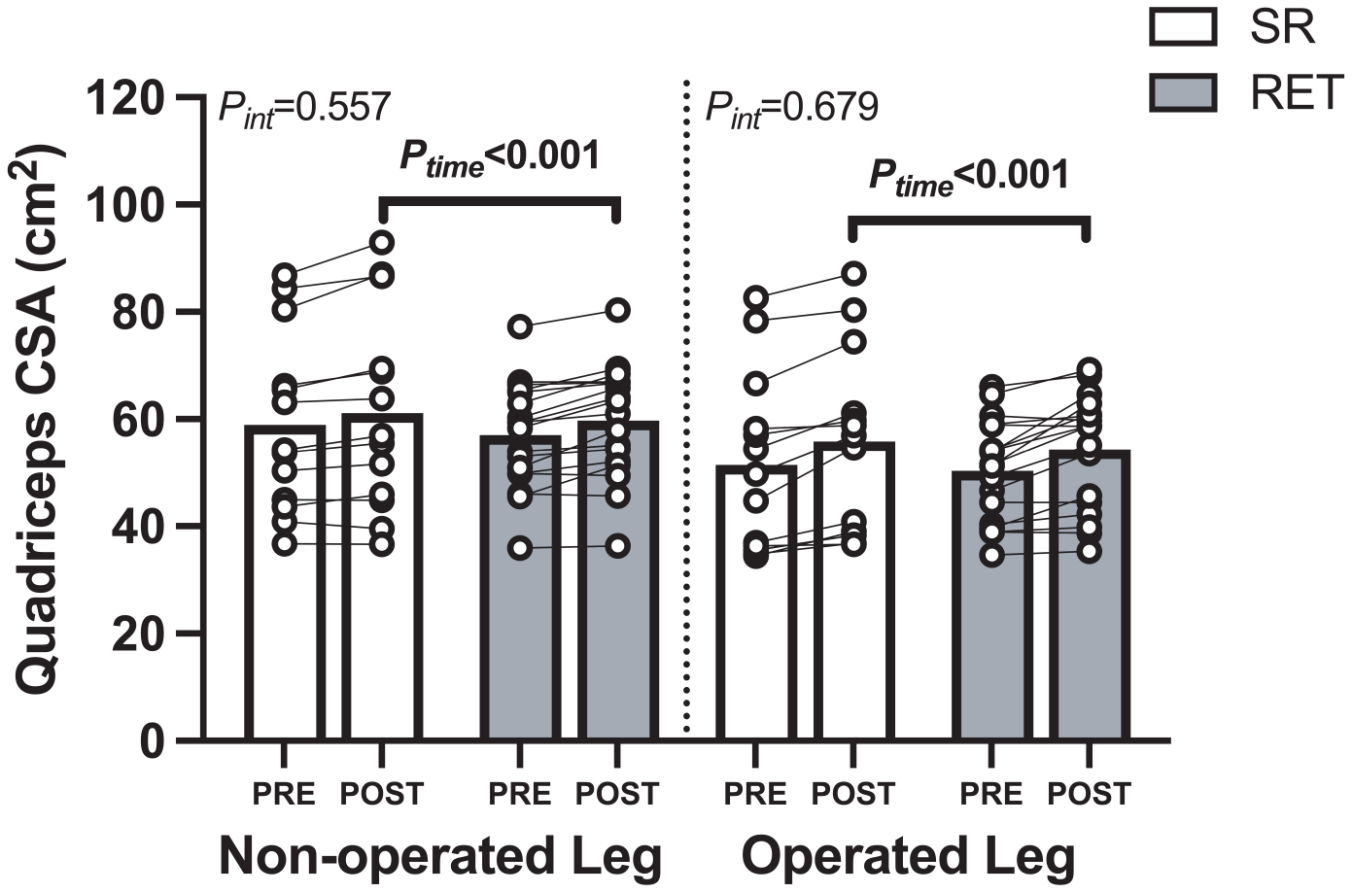
Quadriceps CSA of the nonoperated and operated legs before (pre) and following (post) 12 wk of SR or a RET, which began 8 wk following TKA. Each dot represents data for one participant. Data were analyzed with two-way repeated-measures ANOVAs.

CT data from one participant in the SR group was omitted from analyses owing to deformation of the thigh muscles during the scan; CT data for the remaining participants (SR: *n* = 14, RET: *n* = 18) are presented here. In the operated leg, quadriceps CSA increased in both groups (SR: from 51.4 ± 16.0 cm^2^ to 55.8 ± 16.5 cm^2^, RET: from 50.4 ± 9.45 to 54.3 ± 10.6 cm^2^), with no difference between groups (*P*_time_ < 0.001, η^2^ = 0.60, *P*_int_ = 0.679, η^2^ = 0.01; Figure 3). Similarly, quadriceps CSA in the nonoperated leg increased in both the SR and RET group, with no differences between groups (*P*_time_ < 0.001, η^2^ = 0.54, *P*_int_ = 0.557; η^2^ = 0.01; Figure 3). In line with these findings, whole-thigh muscle CSA increased in both the operated (SR: 113 ± 28 to 119 ± 28 cm^2^, RET: 115 ± 21 to 122 ± 23 cm^2^, *P*_time_ < 0.001, η^2^ = 0.52), and nonoperated leg (SR: 122 ± 29 to 126 ± 31 cm^2^, RET: 123 ± 20 to 128 ± 23 cm^2^, *P*_time_ < 0.001, η^2^ = 0.50), with no differences between groups (*P*_int_ ≥ 0.454, η^2^ ≤ 0.19).

### Muscle Strength

**FIGURE 4:**
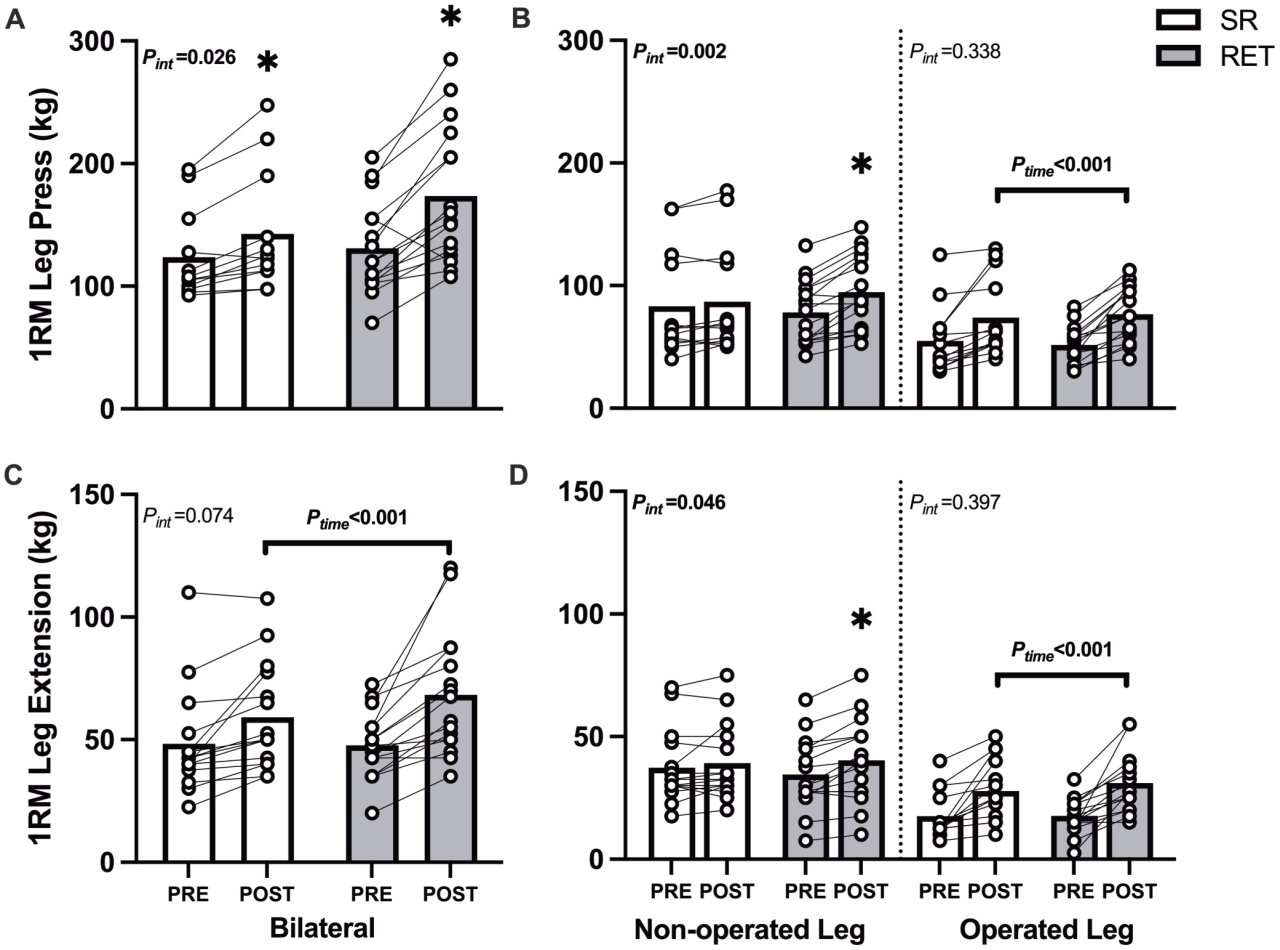
1RM on the leg press (A = bilateral, B = unilateral) and leg extension (C = bilateral, D = unilateral) before (pre) and following (post) 12 wk of SR or a RET, which began 8 wk following TKA. Each dot represents data for one participant. Data were analyzed with two-way repeated-measures ANOVAs. *Significant within-group simple time effect in case of significant interaction effect (*P* < 0.05).

Bilateral and unilateral 1RM data for leg press and leg extension are presented in Figure 4 and Supplemental Table 1, Supplemental Digital Content 1, https://links.lww.com/MSS/D398. Owing to physical constraints (e.g., not able to achieve desired knee angle at baseline), some participants could not perform the 1RM measurements at one or both time points. Therefore, leg press 1RM analyses were performed on 16 RET (*n* = 17 for nonoperated leg) and 13 SR patients (*n* = 12 for bilateral 1RM, as 1RM for one patient was higher than the available machine load). For leg extension 1RMs, data were available for 16 RET and 15 SR patients. Bilateral leg press muscle strength increased significantly over time in both groups; however, the increase was significantly greater (*P*_int_ = 0.026, η^2^ = 0.18) following 12 wk of RET (43 ± 32 kg, *P* < 0.001, η^2^ = 0.62) compared with SR (19 ± 16 kg, *P* = 0.018, η^2^ = 0.20). Bilateral leg extension 1RM also increased significantly over time (RET: 21 ± 17 kg, SR: 11 ± 11 kg, *P*_time_ < 0.001, η^2^ = 0.55); however, there was only a trend toward a difference between groups (*P*_int_ = 0.074, η^2^ = 0.11). When the operated legs were tested separately, both leg press and leg extension 1RM increased similarly for both groups (*P*_time_ < 0.001, *P*_int_ ≥ 0.34). In contrast, significant time*group interaction effects (leg press: *P* = 0.002, leg extension: *P* = 0.046) were observed for the nonoperated leg, with within-group analyses showing that leg muscle strength in the nonoperated leg increased in the RET group only (*P* < 0.001), while it did not change in the SR group (leg press: *P* = 0.175, leg extension: *P* = 0.187).

### Physical Functioning

**FIGURE 5:**
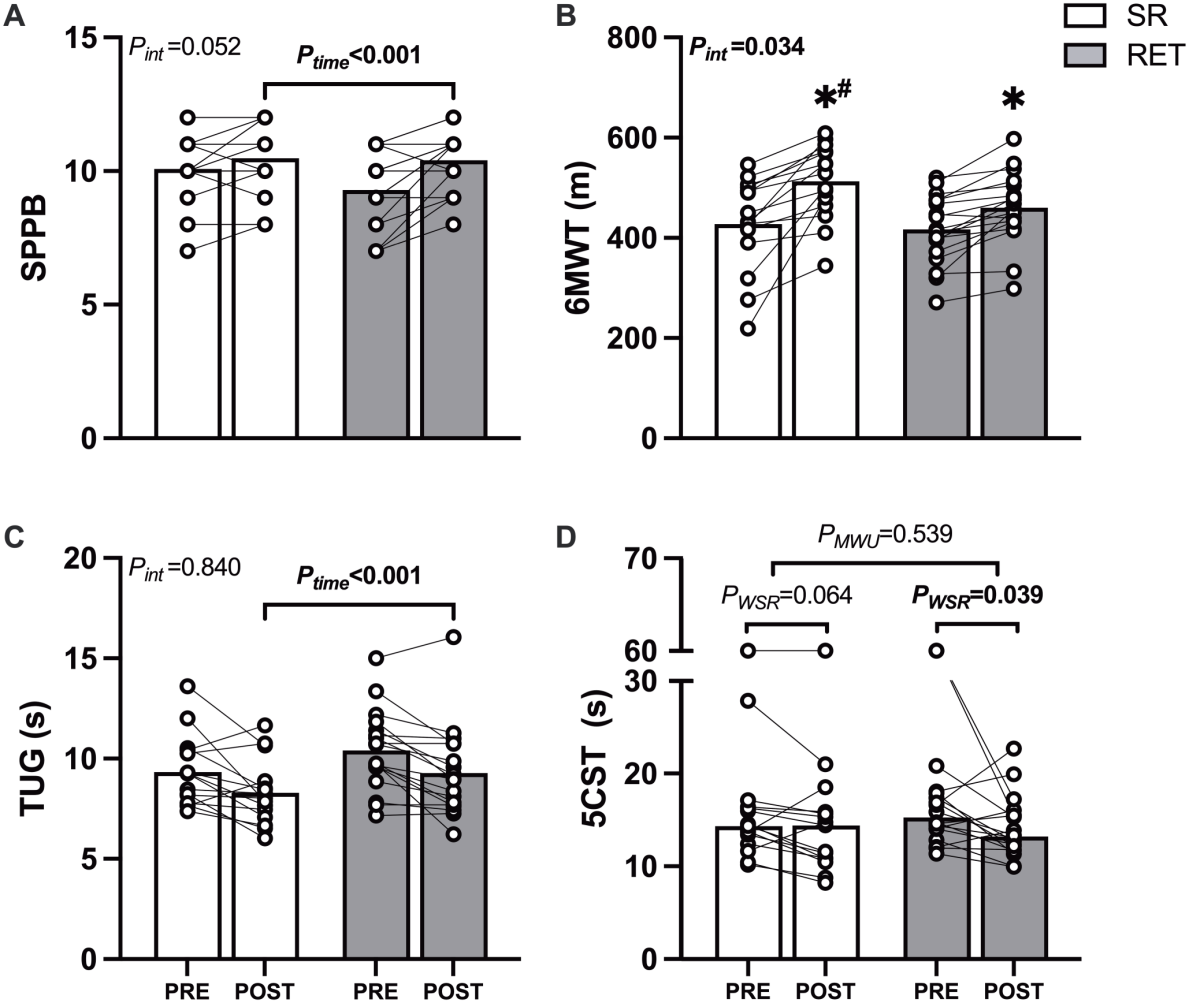
Physical functioning outcomes for the SPPB (A), 6MWT (B), TUG (C), and 5CST (D) before (pre) and following (post) 12 wk of SR or a RET, which began 8 wk following TKA. Each dot represents data for one participant. Data for A–C were analyzed with two-way repeated-measures ANOVAs. For D, bars represent medians, and within-group differences were analyzed using WSR tests, while between-group differences were assessed via a MWU test on the change scores. *P*_int_ = *P* value of interaction. *P*_*time*_ = *P* value of main time effect. MWU, Mann–Whitney *U* test; WSR, Wilcoxon Signed-Rank. *Significant within-group simple time effect (*P* < 0.05), #Significant between-group simple group effect (*P* = 0.049).

Data for the SPPB, TUG, 6MWT, and 5CST are displayed in Figure 5. A tendency for a time*group interaction effect (*P*_int_ = 0.052, η^2^ = 0.12, *P*_time_ < 0.001, η^2^ = 0.37) was observed for the SPPB, which was driven by an improvement over time following 12 wk of RET (from 9.2 ± 1.5 to 10.4 ± 1.1, *P* < 0.001, η^2^ = 0.42), whereas no change was observed in the SR group (pre: 10.1 ± 1.3, post: 10.5 ± 1.4, *P* = 0.134, η^2^ = 0.07). Both groups completed the TUG test faster at the end of the 12-wk intervention period (*P* < 0.001, η^2^ = 0.41), with no differences between the groups (*P*_int_ = 0.840; Figure 5C). While both groups significantly improved in the 6MWT, this improvement was greater (*P*_int_ = 0.034, η^2^ = 0.14) in the SR group compared with the RET group (SR: from 428 ± 94 to 513 ± 75 m, *P* < 0.001, RET: from 417 ± 69 to 460 ± 72 m, *P* = 0.002). Two participants in the RET and one in the SR group could not perform a single repetition of the 5CST at baseline, of which the participant in the SR group also could not perform the test at post-testing. Nonparametric analyses revealed a significant improvement over time in the RET group (from 15.3 [4.2] to 13.2 [3.9] s, *P* = 0.039), while there was only a tendency for a change over time in the SR group (from 14.4 [4.0] to 14.4 [5.1] s, *P* = 0.064). However, no between-group differences were observed on the 5CST change scores (*P* = 0.539).

### Questionnaires

Data from the questionnaires are missing from one participant in the SR group. The remaining participants’ data were analyzed and are presented in Table 3. The total WOMAC score, as well as all subscores, improved significantly over time in both groups (*P* ≤ 0.038), with no differences between the groups (*P*_int_ ≥ 0.573). Similar results were observed for the analyzed scales of the SF-36 and the SF-SarQoL total score, which improved significantly over time in both groups (*P* < 0.001) with no differences between the groups (*P*_int_ ≥ 0.088).

**TABLE 3.** Questionnaire scores before (pre) and following (post) 12 wk of SR or a RET, which began 8 wk following TKA.

		RET (*n* = 17)	SR (*n* = 15)		*P*	
				T*G	T	G
WOMAC						
Total	Pre	31 ± 15	27 ± 14	0.721	**<0.001**	0.357
	Post	23 ± 16	18 ± 15			
Pain	Pre	7 ± 3	4 ± 3	0.573	**<0.001**	**0.046**
	Post	5 ± 5	3 ± 3			
Stiffness	Pre	4 ± 2	4 ± 2	0.668	**0.038**	0.815
	Post	3 ± 2	3 ± 3			
Functioning	Pre	20 ± 11	18 ± 10	0.585	**0.001**	0.500
	Post	15 ± 11	12 ± 11			
SF-36						
Physical functioning	Pre	68 ± 15	56 ± 18	0.088	**<0.001**	0.176
	Post	74 ± 15	70 ± 20			
Role limitations owing to physical health	Pre	48 ± 23	31 ± 14	0.103	**<0.001**	0.140
	Post	69 ± 21	66 ± 24			
Bodily Pain	Pre	35 ± 18	39 ± 21	0.838	**<0.001**	0.443
	Post	61 ± 25	67 ± 26			
SF-SarQoL						
Total	Pre	46 ± 10	42 ± 19	0.981	**<0.001**	0.487
	Post	58 ± 14	54 ± 19			

Data were analyzed with two-way repeated-measures ANOVA and are presented as mean ± SD. *P* values for the time*group interaction effect (T*G) and main effects of time (T) and group (G) are shown.

Significant *P* values are bolded.

### Habitual Dietary Intake and Physical Activity

Physical activity data were complete for 14 SR and 16 RET, and dietary data for 14 SR and 18 RET participants. Devices were worn for a mean of 7 ± 1 and 6 ± 1 days, for 14.5 ± 1.1 and 14.7 ± 1.2 h/d at the pre- and post-time points, respectively, with no significant time*group, *time*, or *group* effects (all *P* > 0.05). There was a significant increase in mean daily activity-related energy expenditure (from 516 ± 211 to 615 ± 270 kcal·d^−1^, *P*_time_ = 0.022, *P*_int_ = 0.109) and total METs (from 1.65 ± 0.22 to 1.71 ± 0.23 METs, *P*_time_ = 0.045, *P*_int_ = 0.332), with no differences between groups. Daily steps also increased significantly over time, but only in the SR group (SR: from 5426 ± 1743 to 7125 ± 3491 steps·d^−1^, *P* = 0.001, RET: 5132 ± 1860 to 5418 ± 1925 steps·d^−1^, *P* = 0.584; *P*_int_ = 0.031).

Total daily energy intake did not change over time or differ between groups (SR: pre: 1986 ± 429, post: 1929 ± 451 kcal·d^−1^; RET: pre: 2133 ± 549, post: 2253 ± 381 kcal·d^−1^; *P*_time_ = 0.146, *P*_int_ = 0.929). However, both absolute and relative protein intake increased significantly in the RET group (from 84 ± 19 to 130 ± 19 g·d^−1^ and from 1.0 ± 0.3 to 1.6 ± 0.3 g·kg BM^−1^·d^−1^, respectively; both *P* < 0.001) but not in the SR group (from 80 ± 22 to 80 ± 14 g·d^−1^, *P* = 0.589, and from 0.9 ± 0.2 to 0.9 ± 0.2 g·kg BM^−1^·d^−1^, *P* = 0.789, respectively), resulting in significant group differences at the postintervention time point (*P* < 0.001, *P*_int_ < 0.001). The increases in protein intake in the RET group were driven by the supplement, as no changes in absolute or relative habitual protein intake were observed when excluding the supplement in the RET group (85 ± 19 g·d^−1^ and 1.0 ± 0.3 g·kg BM^−1^·d^−1^, respectively, *P*_time_ ≥ 0.552, *P*_int_ ≥ 0.830). Excluding the supplement from the analyses did not affect the change over time or difference between groups for total daily energy intake.

## DISCUSSION

The present study investigated the effects of 12 wk of high-intensity resistance exercise training combined with nutritional support compared with SR on muscle strength, muscle mass, and physical functioning in older adults recovering from TKA. Maximal muscle strength improved to a greater extent in the RET compared with the SR group, driven by greater strength gains in the nonoperated leg. However, no between-group differences were observed for strength gains in the operated leg or muscle hypertrophy, and physical functioning outcomes did not consistently favor either condition.

Patients suffering from knee osteoarthritis experience knee pain and reduced joint functioning that causes a decline in physical activity and is ultimately accompanied by strength losses and atrophy in the leg muscles (2). TKA improves much of the osteoarthritis complaints, and following surgery, the declines in muscle mass, strength, and physical function are (partially) reversed. However, previous literature has shown that, despite physical rehabilitation, TKA patients remain with muscle-related impairments even up to a year after surgery (3–5). This setback in muscle-related health outcomes has been associated with suboptimal physical activity and nutritional intake (41,42). Therefore, in the present study, we assessed whether adding a combined intervention of high-intensity resistance exercise training with nutritional support to postoperative rehabilitation would lead to greater improvements in muscle-related outcomes compared with SR alone over the period between 2- and 5-mo post-TKA.

Muscle mass deficits are a common feature in TKA patients and are observed when compared with healthy individuals, as well as when comparing the operated with the nonoperated leg (10). Nonetheless, the effects of physical rehabilitation with or without (progressive) resistance exercise on muscle mass outcomes following TKA are underreported in the literature. Furthermore, resistance exercise studies in TKA patients have generally not taken diet into account, despite accumulating knowledge that the intake of sufficient protein (1.2–1.5 g·kg BM^−1^·d^−1^) and certain micronutrients such as vitamin D and calcium can play an important role in facilitating and optimizing muscle adaptations to exercise training (22–24,26,27). In the present study, DXA and CT scanning were used to assess changes over time in lean mass and muscle CSA, respectively. While the DXA showed no changes over time in leg lean mass, increases in thigh muscle CSA were detected with the CT scans. This discrepancy may be owing to the higher sensitivity and specificity of CT to detect changes in leg muscle size over time (43). The CSA of the quadriceps/thigh muscles increased over time in both legs (operated: ~6%–9%, nonoperated: ~4%–5%) with no differences between the RET and SR groups. Valtonen et al. (13) observed a similar level of hypertrophy of the thigh muscles in the operated leg (5%) in TKA patients that performed 12 wk of (aquatic) resistance training. However, in contrast to our observations, the control group in Valtonen’s study (13) did not show muscle mass gains in the nonoperated leg, and the gains in the operated leg were significantly lower (2%) compared with those in the training group. Although the larger sample size in their study (*n* = 50) may have increased the power to detect between-group differences, other study design features likely explain the discrepancy with the current findings (13). Specifically, the control group in the current study received SR, whereas in Valtonen’s study a control group with no training/treatment was included. Furthermore, the postoperative stage at which the interventions began was standardized at 2 mo in the current study but ranged between 4 and 18 mo post-TKA in their work. Taken together, the findings suggest that, in the earlier stages of TKA recovery, a relatively mild (i.e., low intensity) training stimulus with habitual dietary intake is sufficient to induce hypertrophy.

In line with the muscle mass gains, leg muscle strength also increased in both groups throughout the 12-wk rehabilitation period in the current study. Notably though, bilateral strength gains were much larger in the RET versus the SR group for both the leg press (34% vs 15%) and leg extension (44% vs 27%). Importantly, as participants in the RET group trained regularly using the same exercises on which 1RM assessments were performed, the presence of a learning effect cannot be completely excluded. That said, the magnitude of observed strength gains in the RET group are consistent with previous reports (25%–45%) in untrained older adults following 12 wk of high-intensity resistance exercise training (39,44). Furthermore, all participants completed a standardized 1RM familiarization session at baseline, and some also performed leg press and leg extension exercises during routine physiotherapy in the SR group, albeit at much lower training intensities/frequencies. Hence, the observed group differences are likely owing to truly greater (neuro-)muscular adaptations in the RET versus SR group, rather than familiarity with the testing procedure alone. These results suggest that the inclusion of high-intensity resistance training with targeted nutritional support can induce much larger strength gains than those triggered by SR procedures, which generally involve low-intensity training and little to no attention to nutrition.

Interestingly, while the bilateral strength results were reflected in the nonoperated leg 1RMs, strength gains in the operated leg did not differ between groups. Moreover, these gains in operated leg strength were notably larger than that of the nonoperated leg, with, for example, operated versus nonoperated leg press 1RM increasing by 51% versus 22% in the RET group and 40% versus 6% in the SR group, respectively. These large strength gains in the operated leg versus the nonoperated leg highlight the extent to which the knee extensors were deconditioned in response to surgery and the months-to-years of knee osteoarthritis-associated complaints. However, given that the strength gains in the operated leg in the SR group were beyond what has been reported in other studies (10), there may be other factors inflating these gains. For example, the baseline 1RMs may not have reflected true maximal strength capacity, as postoperative pain and apprehension to load the operated limb likely limited maximal effort at this relatively early postoperative time point (~7 wk postsurgery). Importantly, such factors would increase interindividual variability, thereby reducing power to detect potential between-group differences. Considering the early postoperative limitations in 1RM assessments, it is possible that a longer intervention or follow-up would be required for between-group differences in operated leg strength to become apparent. Alternatively, unilateral strength training may represent a more targeted strategy to preferentially load the operated leg, particularly given that several participants reported consciously offloading the operated limb, relying more on the nonoperated leg during bilateral exercises. Notwithstanding the lack of group differences observed in operated leg strength gains, it is important to note that bilateral muscle strength may be more relevant than unilateral strength for overall functional capacity and maintenance of physical independence in the long term. Therefore, though the differences were mostly driven by the nonoperated leg, we believe that the greater bilateral strength gains observed in the RET versus SR group are clinically meaningful. Future studies should investigate whether the greater bilateral strength responses translate into superior long-term recovery or functional outcomes during TKA rehabilitation.

Improving a patient’s physical functioning is currently the primary aim of post-TKA rehabilitation practices. While a certain level of muscular strength is needed for overall physical functioning, the extent to which strength gains translate to improvements on functional tests during post-TKA recovery is likely dependent on the specific characteristics of that test (10). In the present study, we used a variety of tests to try to capture the limitations in physical functioning relevant to TKA patients (e.g., balance, gait, and leg muscle function). All physical functioning outcomes improved significantly over time during the study period, with no clear advantage for one group over the other. This is further supported by the improvements in physical functioning and quality of life as assessed through various questionnaires, which also did not differ between groups. Only the 6MWT improved to a significantly greater extent in the SR group compared with the RET group. This finding diverges from the literature, in that most individual trials have observed no group differences (15,19,21), while a recent meta-analysis even found greater improvements in 6MWT distance in TKA patients following strength training programs compared with control conditions (45). However, it is possible that the greater number of physiotherapy sessions attended by the SR group in the current study may have resulted in the larger improvements in the walking test, as the main goal of physiotherapy during SR is to regain functioning in everyday tasks. Hence, resistance exercise training alone is not necessarily preferred for regaining physical functioning during early TKA recovery over specific functional exercises during usual care. On the contrary, the 5CST improved significantly in the RET group, while it only tended to improve in the SR group. Likewise, the SPPB (which includes the 5CST), tended to improve more in the RET versus SR group. The 5CST is largely influenced by (bilateral) thigh muscle strength, whereas the 6MWT is more influenced by other factors, such as balance and cardiovascular endurance. Considering that maximal bilateral leg strength improved more in the RET group, this likely translated to greater 5CST improvements as well. These data highlight that physical functioning is influenced by a combination of factors and suggest that TKA rehabilitation requires both lower-intensity, function-focused exercises, as well as higher-intensity, strength-based training with due attention to muscle-supporting nutrition.

It is important to note that, despite encouraging participants in the RET group to also continue with their recommended SR procedures, many expressed that attending an additional three exercise sessions per week was a great time commitment. This commitment to the high-intensity program may have contributed to the lack of increased step count in the RET group, as well as to the RET group attending far fewer physiotherapy sessions than the SR group. These observations likely contributed to the mixed findings in the present study. Therefore, practical implementation would require integrating higher-intensity exercises within existing physiotherapy sessions, and incorporating nutritional support within routine care, rather than applying it as an “add-on” program. Another factor contributing to the current findings is that post-TKA physiotherapy varies greatly in the equipment and type/intensity of exercises performed, as well as level of supervision. As a result, the exact rehabilitation procedure in the SR group was not standardized and could not be precisely quantified. Based on the physiotherapy questionnaire, it was clear that training intensities during supervised physiotherapy in the SR group were relatively low, with some reporting only bodyweight exercises. Additionally, structured nutritional support was not provided in the SR group, clearly distinguishing it from the combined lifestyle intervention in the RET group. Although the lack of standardization in the SR group is inherent to the inclusion of patients from a “real-life” clinical setting, it obviously adds more variation in the data. As a final point of attention, it remains to be established to what extent the participants had already regained some of the muscle mass and strength losses during the first 6–8 wk of post-TKA recovery, as well as to what extent high-intensity resistance exercise training with optimized nutrition may provide long-term benefits beyond the 5-mo post-TKA end point of the current study. That said, the present study provides valuable insight into the changes that occur in muscle outcomes in TKA patients during a critical period of their recovery and demonstrates that the combined approach of high-intensity resistance exercise training with nutritional support augments bilateral strength gains induced during SR.

## CONCLUSIONS

In conclusion, TKA patients experience substantial increases in muscle strength, muscle mass, and physical functioning during the period of 2–5 mo after surgery. Additional high-intensity resistance exercise combined with nutritional support during this period leads to greater gains in bilateral muscle strength than SR alone, mostly driven by greater gains in the nonoperated leg. However, this optimized multimodal intervention does not further enhance operated leg strength, physical functioning, or muscle hypertrophy during TKA recovery. Although high-intensity resistance exercise training with nutritional support has the potential to maximize strength gains, it does not cover all aspects of rehabilitation and, as such, should be carefully integrated within existing paradigms of standard care to achieve optimal recovery.

The authors thank Branco Nijst for helping with patient recruitment and Vita Nova Rehabilitation Centre in Roermond for allowing the use of their training facilities. They thank the participants for their dedication to the study. C.M.S.-P. is an employee of Friesland Campina. L.J.C.v.L., L.B.V., P.G., and L.C.P.G.M.d.G. have received research grants, consulting fees, speaking honoraria, or a combination of these for research on the impact of exercise and nutrition on muscle metabolism, nutrition and aging, and protein quality of plant sources, not related to the current work. The results of the study are presented clearly, honestly, and without fabrication, falsification, or inappropriate data manipulation. The results of the present study do not constitute endorsement by the American College of Sports Medicine. This study was registered at ClinicalTrials.gov (Identifier: NCT05354310).

Data Availability Statement: The datasets generated during and/or analyzed during the current study are not publicly available but are available from the corresponding author upon reasonable request.

Conflict of Interest and Funding Source: This research was supported by funding from the Dutch Research Council (NWO/ZonMW) as well as Friesland Campina.

## Supplementary Material
