# Supplementary material for: High-Intensity Resistance Training Enhances Strength, But Not Muscle Mass and Physical Functioning Gains during Total Knee Arthroplasty Rehabilitation
Source: Med Sci Sports Exerc. 2026 Apr 7;58(8):1651–63. doi: 10.1249/MSS.0000000000004003 (PMC13331270; doi:10.1249/MSS.0000000000004003)
Supplement: Supplementary file 1 [file msse-58-1651-s001.pdf]

Supplemental Table 1. One-repetition maximum tests on leg press and leg extension machines before (PRE) and following (POST) 12 weeks of standard rehabilitations (SR) or a resistance exercise training-based lifestyle intervention with nutritional support (RET), which began 8 weeks following total knee arthroplasty.

												P		
		n	**PRE**			**POST**			**T*G**	**T**	**G**	**T_{WG}**
**Leg Press,** *kg*												
Bilateral	**RET**	*16*	131	±	38	174	±	56	**0.026**			**<0.001**
	**SR**	*12*	124	±	36	143	±	49				**0.018**
Operated leg	**RET**	*16*	52	±	15	77	±	23	0.338	**<0.001**	0.990	
	**SR**	*13*	55	±	28	74	±	32				
Non-operated leg	**RET**	*17*	78	±	25	95	±	30	**0.002**			**<0.001**
	**SR**	*13*	83	±	43	87	±	45				0.175
**Leg Extension,** *kg*												
Bilateral	**RET**	*16*	48	±	14	68	±	25	0.074	**<0.001**	0.555	
	**SR**	*15*	48	±	22	59	±	22				
Operated leg	**RET**	*16*	18	±	7	31	±	12	0.397	**<0.001**	0.608	
	**SR**	*15*	18	±	9	28	±	13				
Non-operated leg	**RET**	*16*	35	±	15	40	±	17	**0.046**			**<0.001**
	**SR**	*15*	37	±	15	39	±	16				0.187

Data were analysed with two-way repeated measures ANOVAs and are presented as mean ± SD. *P*-values for the *time*group* interaction effect (T*G), main effects of time (T) and group (G), and simple within-group time effects (T_{WG}) are shown, where applicable. Significant *P*-values are bolded.
